# Influence of COVID-19 lockdowns on patterns of coercive measures in Austria

**DOI:** 10.1192/j.eurpsy.2023.109

**Published:** 2023-07-19

**Authors:** M. Fellinger

**Affiliations:** Department of Psychiatry and Psychotherapy, Clinical Division of Social Psychiatry, Medial University of Vienna, Vienna, Austria

## Abstract

**Abstract: Background and Aim:**

Coercive measures (CMs), such as involuntary psychiatric admission and mechanical restraint, are considered a last resort in the treatment of people with psychiatric disorders. Although numerous factors influencing its use have been identified, the impact of a pandemic and in particular restrictions like lockdowns on CMs are still unclear. Thus the aim of the present retrospective study was to examine the effects of the COVID-19 pandemic, especially the lockdowns, on CMs in Austria.

**Method:**

This retrospective exploratory study assessed all CMs in Austria, except for the federal state of Vorarlberg, between 01.01.2018 and 31.12.2020. Descriptive statistics and regression models were performed.

**Results:**

During the three-year study period, 40,012 individuals (45.9% females, mean age 51.3 years) had 66,124 involuntary psychiatric admissions for an average of 10.9 days and restraint in 33.9%. In periods of COVID-19 lockdowns (2020 vs. 2018/2019), CMs in form of involuntary admissions were significantly fewer (OR:0.93, p=.0001) but longer (11.6 (SD:16) vs. 10.9 (SD:15.8) days). The likelihood of involuntary admission during lockdowns was only associated with year (2020 vs. 2018/19, p=0.0002), but not with sex (p=0.814), age (p=0.310), use of mechanical restraint (p=0.653) or type of ward (p=0.843).

**Conclusions:**

Restrictions such as lockdowns are affecting CMs and have resulted in fewer but longer involuntary psychiatric admissions during weeks of lockdown in Austria. The result strengthens previous knowledge that showed the dependence on external factors when using CMs, but requires further clarification with regard to the causality and the association with outcomes that are intended to be prevented, such as suicides.

**Disclosure of Interest:**

None Declared

